# Rationale and design of an investigator-initiated, multicenter, prospective open-label, randomized trial to evaluate the effect of ipragliflozin on endothelial dysfunction in type 2 diabetes and chronic kidney disease: the PROCEED trial

**DOI:** 10.1186/s12933-020-01065-w

**Published:** 2020-06-13

**Authors:** Atsushi Tanaka, Michio Shimabukuro, Yosuke Okada, Kazuhiro Sugimoto, Akira Kurozumi, Keiichi Torimoto, Hiroyuki Hirai, Koichi Node, the PROCEED trial investigators

**Affiliations:** 1 Department of Cardiovascular Medicine, Saga University, Saga, Japan; 2 Department of Diabetes, Endocrinology, and Metabolism, Fukushima Medical University, Fukushima, Japan; 3 The First Department of Internal Medicine, School of Medicine, University of Occupational and Environmental Health, Kitakyushu, Japan; 4 Diabetes Center, Ohta Nishinouchi Hospital, Koriyama, Japan; 5 Wakamatsu Hospital of the University of Occupational and Environmental Health, Kitakyushu, Japan; 6 Department of Internal Medicine, Shirakawa Kosei General Hospital, Shirakawa, Japan

**Keywords:** Chronic kidney disease (CKD), Endothelial dysfunction, Ipragliflozin, Reactive hyperemia peripheral arterial tonometry (RH-PAT), Sodium glucose cotransporter 2 inhibitor (SGLT2i), Type 2 diabetes (T2D)

## Abstract

**Background:**

Type 2 diabetes (T2D) is associated with renal impairment and vascular endothelial dysfunction. Therefore, this pathological connection is an important therapeutic target. Recent cardiovascular and renal outcome trials demonstrated that sodium glucose cotransporter 2 inhibitors (SGLT2is) consistently reduced the risks of cardiovascular and renal events and mortality in patients with T2D and various other background risks including chronic kidney disease (CKD). However, the precise mechanisms by which SGLT2is accords these therapeutic benefits remain uncertain. It is also unknown whether these SGLT2is-associated benefits are associated with the amelioration of endothelial dysfunction in patients with T2D and CKD.

**Methods:**

The PROCEED trial is an investigator-initiated, prospective, multicenter, open-label, randomized-controlled trial. The target sample size is 110 subjects. After they furnish informed consent and their endothelial dysfunction is confirmed from their decreased reactive hyperemia indices (RHI), eligible participants with T2D (HbA1c, 6.0–9.0%) and established CKD (30 mL/min/1.73 m^2^ ≤ estimated glomerular filtration ratio [eGFR] < 60 and/or ≥ urine albumin-to-creatinine ratio 30 mg/g Cr) will be randomized (1:1) to receive either 50 mg ipragliflozin daily or continuation of background treatment (non-SGLT2i). The primary endpoint is the change in RHI from baseline after 24 weeks. To compare the treatment effects between groups, the baseline-adjusted means and their 95% confidence intervals will be estimated by analysis of covariance adjusted for HbA1c (< 7.0% or ≥ 7.0%), age (< 70 y or ≥ 70 y), RHI (< 1.67 or ≥ 1.67), eGFR (< 45 mL/min/1.73 m^2^ or ≥ 45 mL/min/1.73 m^2^), and smoking status. Prespecified responder analyses will be also conducted to determine the proportions of patients with clinically meaningful changes in RHI at 24 weeks.

**Discussion:**

PROCEED is the first trial to examine the effects of ipragliflozin on endothelial dysfunction in patients with T2D and CKD. This ongoing trial will establish whether endothelial dysfunction is a therapeutic target of SGLT2is in this population. It will also provide deep insights into the potential mechanisms by which SGLT2is reduced the risks of cardiovascular and renal events in recent outcome trials.

*Trial registration* Unique Trial Number, jRCTs071190054 (https://jrct.niph.go.jp/en-latest-detail/jRCTs071190054).

## Background

Type 2 diabetes mellitus (T2D) is often associated with microvascular complications, such as impaired kidney function, and is a major cause of chronic kidney disease (CKD) and end-stage renal disease (ESRD) [1, 2]. CKD is one of the strongest established risk factors for cardiovascular events and increased mortality [3, 4]. Over the past two decades, intensive blood glucose control and interventions targeting multiple risk factors have been recognized as established therapeutic strategies to reduce the risk of microvascular complications and improve outcome [5, 6]. Renin-angiotensin-aldosterone system (RAAS) blockade is the only medication approved for preventing decline in kidney function and development of ESRD in patients with T2D [7, 8]. Nevertheless, many people diagnosed with T2D still present with CKD and ESRD [9] and, by extension, an important residual risk of worsened health outcomes. Therefore, the development of effective supplementary renoprotective therapies for patients with T2D is urgently required.

A sodium glucose co-transporter 2 inhibitor (SGLT2i) was formulated to reduce blood glucose by blocking glucose reabsorption at the renal proximal tubule. Recent cardiovascular outcome trials (CVOT) demonstrated that SGLT2is improved cardiorenal outcomes in a wide range of patients with T2D [10, 11]. SGLT2is also consistently prevented decline in renal function and reduced CKD progression in the aforementioned CVOTs [12–14]. The CREDENCE trial showed that canagliflozin markedly reduced the risks of composite renal events and mortality in patients with T2D and established CKD [15]. A systematic review and meta-analysis of pooled data from four CVOTs revealed that SGLT2is reduced the risk of composite renal events across all studies irrespective of the baseline levels of renal function, such as estimated glomerular filtration ratio (eGFR) and albuminuria, and baseline RAAS blockade use [16]. The available evidence indicates that these renal effects may be a class effect of SGLT2is [17, 18]. Hence, SGLT2is have become the guideline-recommended treatment option for patients with T2D who are at high risk of cardiovascular events and renal insufficiency [19, 20].

**Fig. 1:**
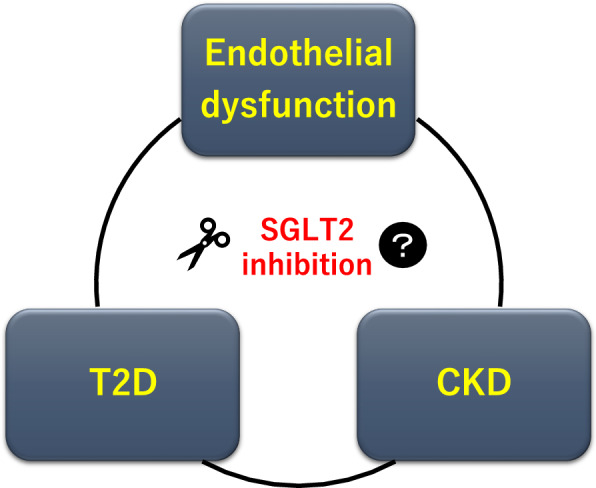
Rationale for the PROCEED trial. Endothelial dysfunction is closely associated with T2D and CKD and forms a pathological triangle. The objective of our trial is to test the hypothesis that SGLT2-mediated inhibition breaks this triangle by ameliorating endothelial dysfunction. Another aim is to elucidate the mechanisms by which SGLT2 inhibitors confer cardiovascular and renal benefits in patients with T2D and CKD. *CKD*: chronic kidney disease; *SGLT2*: sodium-glucose cotransporter 2; *T2D*: type 2 diabetes

To date, various mechanisms have been proposed to explain the renoprotective efficacy of SGLT2is. One plausible theory is that they modulate renal hemodynamics by correcting intraglomerular hyperfiltration [21–24], independent of their glucose-lowering effect [25]. However, the underlying mechanisms responsible for the cardiorenal outcomes of SGLT2i therapy and the influences of these agents on other drivers remain to be elucidated. Thus, we are currently focusing on the effects of SGLT2is on endothelial function in patients with T2D and CKD. Endothelial function may be impaired by various metabolic disturbances, such as insulin resistance and diabetes [26]. Elevated markers indicative of endothelial dysfunction are associated with T2D [27–29]. Endothelial dysfunction is also associated with renal impairment and CKD pathogenesis [30, 31]. Therefore, endothelial dysfunction plays pivotal roles in the development of the pathogenic triangle of T2D and CKD (Fig. 1). The PROCEED trial was designed to test the hypothesis that SGLT2is can break this triangle. Here, we will assess the therapeutic efficacy of SGLT2is on endothelial dysfunction in the attempt to clarify the mechanisms by which SGLT2is confers cardiovascular and renal benefits in patients with T2D and CKD.

## Methods

### Trial overview and design

The Program of Ipragliflozin for Endothelial Dysfunction in Chronic Kidney Disease and Type 2 Diabetes (PROCEED) trial is an ongoing, investigator-initiated, prospective, multicenter, open-label, randomized-controlled trial. Informed consent will be received from eligible subjects and they will be randomized into ipragliflozin or non-SGLT2is use (control) groups. Each treatment regimen will be followed for 24 mo. The effects of ipragliflozin on endothelial dysfunction in comparison to the control will be evaluated by RH-PAT.

Local certified review boards (CRB) approved the trial protocol (No. C20190201). The trial must be conducted in full compliance with the articles of the Declaration of Helsinki and in accordance with the Clinical Trial Act in Japan. The PROCEED trial was entered into the Japan Registry of Clinical Trials (ID: jRCTs071190054) and is funded by Astellas Pharma Inc. However, this funding agency has no role in trial design or execution.

### Trial population and recruitment

A total of 110 participants will be recruited across six sites in Japan. Trial recruitment began in March 2020 and is expected to end by August 2021. Eligible participants are patients aged ≥ 30 y who meet the inclusion and exclusion criteria detailed in Table 1. Eligible patients include those with a diagnosis of T2D, a HbA1c in the range of 6.0–9.0%, on stable glucose-lowering agents for ≥ 3 mo before providing consent, and presenting with established CKD (30 mL/min/1.73 m^2^ ≤ eGFR < 60 and/or ≥ urine albumin-to-creatinine ratio (UACR) 30 mg/g Cr). However, they must have no history of clinically apparent atherosclerotic cardiovascular diseases such as coronary artery disease, stroke, peripheral artery disease, and symptomatic carotid artery stenosis. Based on the recently proposed value for the RH-PAT-derived reactive hyperemia index (RHI) distinguishing normal and impaired endothelial function, RHI ≥ 2.10 is a plausible marker of normal endothelial function [32]. Subjects with RHI < 2.10 at the time of pre-testing RH-PAT ≤ 3 mo prior to randomization were eligible for the present trial. In this way, subjects with normal endothelial function were excluded. After initial eligibility screening using prior medical records, each patient will receive an adequate explanation of the trial plan before they are asked to provide written informed consent.

**Table 1:** Comprehensive inclusion and exclusion criteria

Inclusion	Exclusion
Adults (aged ≥ 30 years) T2D with HbA1c ≥ 6.0% and < 9.0% Pre-existing CKD with (i) 30 mL/min/1.73 m^2^ ≤ eGFR < 60 mL/min/1.73 m^2^ and/or (ii) albuminuria ≥ 30 mg/g Cr Patients with RHI < 2.10 at pre-testing RH-PAT within 3 mo prior to randomization Patients who received an explanation of the study and provided written informed consent	Type 1 diabetes History of clinically apparent atherosclerotic CVDs such as CAD, stroke, PAD, and symptomatic carotid artery stenosis History of arterial fibrillation History of severe ketosis, diabetic coma, or precoma attack ≤ 6 mo prior to informed consent Severe renal dysfunction (eGFR < 30 mL/min/1.73 m^2^ or undergoing dialysis) Patients received SGLT2is within 3 mo before informed consent Patients who changed the glucose-lowering medications within 3 mo before informed consent Hypersensitivity to ipragliflozin or other SGLT2is Symptomatic hypotension, or systolic blood pressure < 90 mm Hg Patients with severe infection or trauma at screening Patients in perioperative period around screening Polysystic kidney disease, lupus nephritis, or ANCA-related vasculitis Pregnant or suspected pregnancy Considered inappropriate for the study by investigators due to other reasons such as malignancy

*ANCA* antineutrophil cytoplasmic antibody; *CAD* coronary artery disease; *CVDs* cardiovascular diseases; *CKD* chronic kidney disease; *eGFR* estimated glomerular filtration rate; *PAD* peripheral artery disease; *RHI* reactive hyperemia index; *RH*-*PAT* reactive hyperemia peripheral arterial tonometry; *SGLT2is* sodium glucose cotransporter 2 inhibitors; *TIA* transient ischemic attack; *T2D* type 2 diabetes

### Trial design and follow-up

**Fig. 2:**
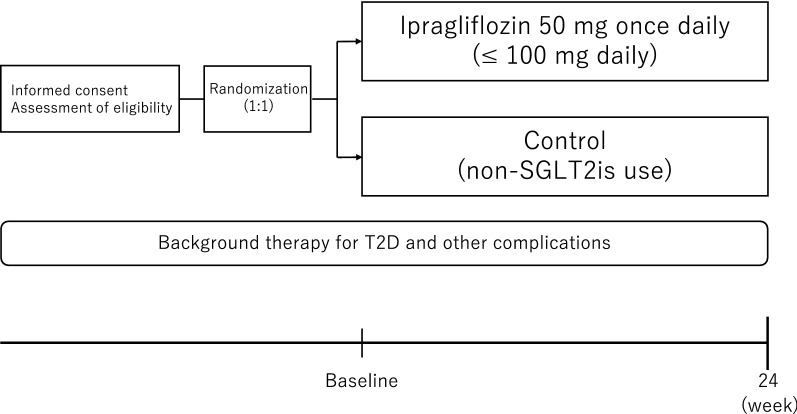
Trial design. *SGLT2is*: sodium-glucose cotransporter 2 inhibitors; *T2D*: type 2 diabetes

All consenting and eligible participants will be randomized and assigned either to the ipragliflozin group or the non-SGLTis use (control) group. There will be a follow-up visit to measure the study endpoints either at 24 weeks or at discontinuation (Fig. 2). However, all participants will visit their regular physicians at unprescribed time points in order to receive usual care, individualized background treatment, administration of the study drug, and monitoring of safety information and drug adherence during the study period.

### Randomization

All participants who signed informed consent and met the enrollment criteria will be randomly assigned (1:1) either to the ipragliflozin treatment or the non-SGLT2is (control) group (Fig. 2). Treatments will be assigned via a web-based program, the minimization method, and biased coin assignment balancing by age (< 70 y; ≥ 70 y), HbA1c level (< 7%; ≥ 7%), current smoking (yes, no), pre-testing RHI (< 1.67; ≥ 1.67), and eGFR (< 45 mL/min/1.73 m^2^; ≥ 45 mL/min/1.73 m^2^) at the time of screening.

### Treatment

All participants will be followed for 24 months. No specific numerical glycemic control target such as the HbA1c level was set for this study. Nevertheless, all participants must be treated in order to achieve a personalized goal recommended by the Japanese treatment guideline [33, 34]. Participants assigned to the ipragliflozin group will receive 50 mg ipragliflozin once daily in addition to their background therapy. If the personalized goal is not achieved, the ipragliflozin dose may be increased by the investigators to 100 mg once daily. Participants assigned to the control group will continue their background therapy. Within the appropriate range of the therapeutic goal, participant background therapy will not change during the study. However, if participants cannot reach their glycemic target, co-administration of antidiabetic agents other than SGLT2 inhibitors or increased dosages of other antidiabetic agents may be considered by the investigators. If the blood glucose level is > 13.3 mmol/L (> 240 mg/dL) after an overnight fast and is confirmed by a second measurement on a different day, rescue medication may be initiated for hyperglycemia treatment. The initiation, choice, and dosage of the rescue medication(s) are left to the discretion of the investigator. Rescue medication may include an up-titration of a background treatment. If insulin is a background therapy, a change of > 10% of the total daily prescribed insulin dose is considered rescue therapy. All rescue medication must be taken in accordance with the prescribing information in order to minimize the risks of contraindications and hypoglycemia. Upon trial completion, participants may select any glucose-lowering treatment in accordance with their individual requirements.

### Measurements

The baseline characteristics of all participants including gender, age, body height, body weight (BW), abdominal circumference, office blood pressure (BP), pulse rate (PR), duration of T2DM, complications, background treatment, and smoking habit will be recorded prior to randomization. The background treatment and trial medication and adherence will be recorded for each patient at each visit. Abdominal circumference, BW, BP, and PR will be measured at baseline and at 24 weeks. The following assessments and methodology will be also applied at baseline and at 24 weeks: peripheral endothelial function according to the RH-PAT test, echocardiography (left ventricular ejection fraction determined by a modified Simpson method, left atrial volume index, E, septal e’, lateral e’, and E/e’ by tissue Doppler imaging), and non-contrast abdominothoracic computed tomography (CT) to measure pericardiac, hepatic, and perirenal fatty tissue, renal size, and blood and urine samples. (Please see Additional file 1). Certain serum biomarkers including *N*-terminal pro-brain natriuretic peptide, erythropoietin, cystatin C, and procollagen III peptide will also be measured at the central laboratory of SRL Inc., Tokyo, Japan. Images of non-contrast abdominothoracic CT will be measured at the core laboratory of the Department of Diabetes, Endocrinology, and Metabolism, Fukushima Medical University, Fukushima, Japan.

### Measurement of RH-PAT

Peripheral endothelial function will be evaluated by RH-PAT using an Endo-PAT2000 device (Itamar Medical, Caesarea, Israel). RH-PAT principles and procedures have been described elsewhere [35–37]. In brief, the measurements will be performed in the mornings at baseline and at 24 wks in a quiet, light- and temperature-controlled room. The patients will be fasted and in stable condition before taking their daily medication. A BP cuff will be placed on the upper arm of the patient and the opposite arm will serve as a control. The PAT probes will be placed on one finger of each hand. The patient will be allowed to rest for ≥ 15-min on a bed in a supine position and his or her baseline pulse amplitude will be recorded from each fingertip for 6 min. The BP cuff will be inflated to 60 mmHg above systolic BP or to 200 mmHg for 5 min. The cuff will then be deflated and the pulse amplitude will be recorded for 5 min. The RHI will be automatically calculated in an operator-independent manner by a computerized algorithm. The augmentation index (AI), heart rate variability (HRV), standard deviation of the normal to normal intervals (SDNN), and the ratio of low-to high-frequency (LF/HF) will be automatically calculated using Endo-PAT2000 software (v. 3.4.4; Itamar Medical, Caesarea, Israel).

### Trial endpoints

The primary endpoint in this trial is the change in natural log-transformed RHI (LnRHI) from baseline to 24 weeks or discontinuation. The secondary endpoints include (i) the  % change in LnRHI from baseline to 24 weeks or discontinuation, (ii) the proportion of patients with clinically meaningful changes in LnRHI (≥ 15% increase from baseline at 24 weeks or discontinuation), (iii) the proportion of patients with clinically meaningful improvement in RHI (≥ 2.10 at 24 weeks or discontinuation), (iv) the proportion of patients attaining (ii) and/or (iii), and (v) the proportion of patients with clinically meaningful changes in LnRHI (≥ 15% decrease from baseline at 24 weeks or discontinuation). Additional efficacy endpoints include changes from baseline to 24 weeks or discontinuation in (i) blood and urine laboratory values (Additional file 1), (ii) vital signs including abdominal circumference, BP, PR, BW, and body mass index, (iii) vascular functional parameters (AI, HRV, SDNN, and LF/HF) measured by a RH-PAT device, (iv) left ventricular systolic and diastolic function measured by echocardiography, and (v) pericardiac, hepatic, and perirenal fatty tissue and renal size measured by non-contrast abdominothoracic CT.

### Safety

Throughout the study, safety information will be collected for the intention-to-treat population by recording serious adverse events (AE) regardless of their causal relationships to the trial drugs and protocol. Upon confirmation of these AEs, the investigators will assess their severity or grade, the procedures conducted, the outcomes, and the relationships to the study drug. Investigators will immediately report AEs to the secretariat who, in turn, will promptly report to them to the principal investigator. In accordance with the Clinical Trial Act in Japan, the principal investigator must report all AEs suspected of having causal relationships with the study to the CRB at Saga University. The CRB will then independently evaluate trial safety, determine the requirement for revisions to the trial design, and validate all decisions to continue the trial. The criteria governing trial withdrawal are listed in Table 2.

**Table 2:** Discontinuation criteria

Participant withdrawal of consent
Serious violation of study protocol
Considered inappropriate to continue the trial by investigators due to following conditions:
Severe hypoglycemia
Development of diabetic ketoacidosis
Serious dehydration requiring rehydration therapy
Seriously poor glycemic control (HbA1c ≥ 10.0%)
Aggravation of primary disease or complications
Adverse side effects of the trial drug
Other reasons
Discontinuation of ipragliflozin or use of other SGLT2is in the ipragliflozin group
Use of SGLT2is in the control group
Serious violation of study protocol
Participant withdrawal of consent

*SGLT2is* sodium glucose cotransporter 2 inhibitors

### Statistical considerations

#### Sample size estimation

The effects of SGLT2is on peripheral endothelial function remain to be elucidated as the currently available evidence is very limited. Sugiyama et al. [38] used RH-PAT to assess the effects of the SGLT2 inhibitor dapagliflozin on endothelial function in Japanese patients with uncontrolled T2D (baseline HbA1c, 7.9%; baseline eGFR, ~ 74 mL/min/1.73 m^2^; unknown cardiovascular event history). The sample size was estimated to detect mean differences of 0.15 and 0.05 in the change in LnRHI for the dapagliflozin and non-SGLT2 inhibitor group, respectively. The estimated group difference was 0.10, the standard deviation was 0.15, the power was 90%, and the two-sided α was 0.05. As a result, 6 mo of dapagliflozin treatment significantly improved endothelial function. The actual difference between the dapagliflozin and non-SGLT2 inhibitor groups in terms of LnRHI was 0.139. On the other hand, the aim of the present trial is to evaluate the effects of ipragliflozin on the aforementioned endpoint in patients with CKD. Compared to those in the study population of Sugiyama et al., these individuals may present with more advanced cardiovascular damage that is difficult to ameliorate using the available interventions. Thus, we estimated a difference of 0.10 ± 0.15 between the groups in terms of the change in LnRHI. At α = 5% for a two-sided test, a sample size of 98 patients was needed to provide a power of 90% for each comparison. Considering a potential dropout rate of 12%, it was estimated that ≥ 110 patients (55 patients per arm) would provide sufficient statistical power for the trial.

#### Statistical analysis plan

Summary statistics will be used to calculate all baseline characteristics including the categorical variable frequencies and proportions and the normally distributed mean ± standard deviation or median [interquartile range] with skewed distribution for the continuous variables. Patient characteristics will be compared by Fisher’s exact test for categorical variables and the Wilcoxon rank sum test for continuous variables.

Analyses of the primary and secondary endpoints will be performed for the full analysis set (FAS). The FAS excludes all participants with no post-randomization efficacy endpoint data and those deemed ineligible after registration because they failed to provide informed consent or did not meeting the study eligibility criteria.

The primary endpoint was a comparison of treatment effects between treatment groups. The baseline-adjusted least-square means (LSM) and their 95% confidence intervals (CI) will be estimated by analysis of covariance (ANCOVA) for the FAS. For a secondary analysis of the primary endpoint, an analysis of variance including allocation factors and treatment group as factors will also be performed. As a supplementary analysis, the primary endpoint will also be analyzed in the per protocol set excluding subjects seriously violating the trial protocol. Prespecified subgroups stratified according to clinical characteristics based on, but not limited to, the RHI at baseline (< 1.67 or ≥ 1.67) will be analyzed for the primary endpoint as described above.

For the secondary endpoints, (i) the % change in LnRHI from baseline to 24 wks or discontinuation will be analyzed by a two-sample *t* test. Their LSMs and 95% CIs will be calculated and the treatment effects will be compared between groups. (ii) The proportions of patients presenting with clinically meaningful changes in LnRHI or RHI will be analyzed with a logistic regression model adjusted by the corresponding baseline values to calculate the odds ratios and their 95% CIs and compare the treatment effects between groups.

Changes in the additional efficacy endpoints from baseline to 24 weeks or discontinuation will be analyzed by ANCOVA as for the primary endpoint. The LSMs and their 95% CIs will be calculated and the treatment effects will be compared between groups. Pearson’s correlation coefficients will be used to identify the correlations between the changes in RHI from baseline and each of the additional efficacy endpoints.

The principal investigator and a biostatistician will confirm the detailed statistical analysis plan before the database is locked. All *p* values will be two-sided. *P* < 0.05 will be considered statistically significant. No adjustments for multiple comparisons will be regarded. All statistical analyses will be performed in SAS v. 9.4 (SAS Institute, Cary, NC, USA).

### Trial organization and oversight (Additional file 2)

The principal investigator of the PROCEED trial is Koichi Node of the Department of Cardiovascular Medicine at Saga University. The trial secretariats are located at the Department of Cardiovascular Medicine of Saga University and the Translational Research Center for Medical Innovation in Kobe, Japan. Data management, monitoring activities, statistical analyses, and audits will be independently implemented in compliance with an outsourcing agreement. Data monitoring will be enforced to ensure proper research performance. An independent audit team will inspect several main institutes to ensure trial data quality.

## Discussion

The PROCEED trial is an investigator-initiated, ongoing, prospective, multicenter, open-label, randomized-controlled clinical trial. Its purpose is to test the hypothesis that ipragliflozin is an effective therapy for T2D patients with established CKD and impaired endothelial function. The primary endpoint of the trial is the change in the peripheral endothelial functional parameter for the treatment group compared to the non-SGLT2is-use control group. This parameter will be measured by RH-PAT from baseline to 24 wks or discontinuation. Several cardiorenal and metabolic markers will also be investigated in this study. In this way, the potential mechanistic association between the changes induced by ipragliflozin treatment and endothelial function may also be evaluated. The present trial may provide novel insights into the pathological role of peripheral endothelial dysfunction in patients with T2D and established CKD and clarify the therapeutic role of ipragliflozin in this process.

Recent cardiovascular outcome trials (CVOTs) involving SGLT2is clearly and consistently associated SGLT2is with substantial reductions in cardiovascular and renal events in patients with T2D and at high risk for these complications. A systematic review and meta-analysis of the three first CVOTs (EMPA-REG OUTCOME, CANVAS Program, and DECLARE-TIMI 58) [11] disclosed that risk reduction provided by SGLT2is for major adverse cardiovascular events (MACE) was significant in a subgroup with a history of atherosclerotic cardiovascular diseases (CVD). However, SGLT2is did not significantly reduce these risks in a subgroup with multiple risk factors. Conversely, SGLT2is was associated with the reduction of risk of composite renal outcomes regardless of CVD history. Moreover, SGLT2is treatment significantly reduced the risk of the aforementioned adverse events in patients with decreased eGFR (< 60 mL/min/1.73 m^2^). Thus, SGLT2is may have renoprotective efficacy for a wide range of T2D patients. The CREDENCE trial was the first to investigate the effects of canagliflozin on composite renal outcome in patients with T2D and established CKD. It confirmed that canagliflozin treatment consistently reduced the development of primary renal composite outcome independent of CVD history and across baseline levels of markers of deteriorating renal function such as decreased eGFR and abnormal albuminuria [15, 39]. Renal benefits in T2D patients with concomitant CKD irrespective of baseline RAAS inhibitors were also reported in several subsequent studies [16, 40, 41]. Therefore, SGLT2is may have class and additive renal efficacy. Giugliano et al. [40] conducted a meta-analysis on data compiled from four CVOTs and found that SGLT2is was more effectively at preventing MACE in subgroups with reduced eGFR (< 60 mL/min/1.73 m^2^) than in those with preserved eGFR (≥ 60 mL/min/mL/1.73 m^2^). Hence, SGLT2is treatment may play an important therapeutic role in patients with T2D and CKD. Further, deteriorated renal function may be a clinically meaningful indicator of the cardiovascular and renal effects of SGLT2is treatment [18].

SGLT2is have multifactorial effects on non-glycemic parameters associated with primary glycosuria and natriuresis. SGLT2is modulates hemodynamics and remodels metabolism. In turn, these responses have cardiovascular and renal benefits [42, 43]. The most widely accepted mechanism of the renoprotective efficacy of SGLT2is is hemodynamically-mediated restoration of tubuloglomerular feedback [21–24, 44]. Several clinical and preclinical trials suggested that SGLT2is correct pathological interconnections between the heart and kidneys. They may be directly renoprotective by reducing inflammation and oxidative stress, attenuating hyperactivated sympathetic nervous systems, and improving vascular function [45, 46].

Endothelial function plays a vital role in maintaining vascular homeostasis but it can be impaired by various metabolic disturbances and systemic diseases [26]. Endothelial dysfunction is implicated in the initial steps of atherogenic change and subsequent atherosclerosis progression. It is associated with cardiovascular events and poor prognosis [27, 47, 48]. The intrarenal endothelium regulates renal microcirculation and its structures. Its dysfunction is also associated with renal damage and CKD progression [30, 31, 49, 50]. Thus, endothelial dysfunction may be linked to both T2D and CKD (Fig. 1). It is, therefore, expected that the cardiovascular and renal benefits of SGLT2is are mediated at least in part by the improvement of endothelial dysfunction, particularly in patients with T2D and established CKD.

Assessment of peripheral endothelial function may furnish clinically useful information on the present cardiovascular risk status, the future outcome, and the evaluation of treatment efficacy [51]. To date, several animal studies have already demonstrated that SGLT2is, including ipragliflozin, ameliorated endothelial dysfunction and vascular and kidney injuries [52–57]. Several clinical studies also investigated the effects of SGLT2is on endothelial function in diabetic patients with and without additional cardiovascular event risk factors [38, 58–64]. However, the results were partially inconsistent. In certain studies, SGLT2is conferred beneficial effects on vascular function in diabetic patients at relatively low risk of cardiovascular events. Nevertheless, there was no obvious effect of 6 months of SGLT2is treatment in patients with established CVDs [62, 63]. Thus, short-term interventions may be too brief to improve endothelial function in patient subpopulations whose vascular injuries are more advanced than those with no CVD history. In the CANVAS, DECLARE-TIMI 58, and CREDENCE trials on patients with T2D and established CKD, SGLT2is treatment consistently improved cardiac- and renal failure outcomes in patients receiving primary prophylaxis [15, 65, 66]. Therefore, SGLT2is may have a therapeutic impact on the cardiorenal axis even in the aforementioned patient subpopulation. Moreover, the improvement of endothelial dysfunction may play a mechanistic role in this process. To the best of our knowledge, however, no previous study has assessed the effects of SGLT2is on vascular parameters such as endothelial function in diabetic patients with established CKD. It has not yet been established whether the beneficial effects of SGLT2is on endothelial dysfunction constitute a mechanism for the robust improvement observed in the patient populations participating in the previous CVOTs. There are ongoing clinical trials testing the hypothesis that SGLT2 inhibitors prevent CKD progression in patients with pre-existing CKD [67, 68]. The present study may also furnish mechanistic insights into the renoprotective efficacy of SGLT2 inhibitors.

This trial had several limitations that were also inherent to similar earlier investigations performed by our study team [37, 69]. First, we did not use a double-blind placebo-controlled trial here but rather an open-label design. This configuration might create an unexpected bias towards assessing potentially subjective endpoints associated with the treatments selected by the investigators. This bias could occur despite the strict requirement that the background medications of all participants must not change throughout the study. Second, the trial sample size is relatively small and the study treatment period is comparatively short. Third, the ANCOVA is set as the primary analysis based on our preliminary presumption of a negative correlation between the LnRHI values and their changes from baseline. We did not establish the correlation coefficient required to calculate the sample size. Hence, we estimated the sample size via a conservative two-sample *t*-test. Fourth, although the RH-PAT examination is non-invasive, easy to perform, and operator-independent, its output is sensitive to individual physical conditions and the ambient environment. To minimize these influences across all participating institutions, investigators must perform the RH-PAT examination in accordance with the operating manual provided by the manufacturer. Further, the present study excludes patients with RHI ≥ 2.10 according to the RH-PAT test performed prior to randomization. This threshold was previously proposed for normal endothelial function. Its border zone was ≥ 1.6 and < 2.10 [32]. The widely accepted cutoff value of RHI is 1.67 for the discrimination of normal and abnormal endothelial function [36]. Thus, changes in RHI in response to the treatments in the present study may differ depending on the baseline level. Therefore, we will attempt to assess the relative impacts of these effects using subgroup analyses as the primary endpoints and responder/non-responder analyses as the secondary endpoints. Finally, as SGLT2is are pharmacologically active in the kidney, their glucose-lowering effect may be insufficient in patients with lower eGFR [70]. However, the renoprotective efficacy of SGLT2is may be independent of glycemic control [25]. At this time, ipragliflozin is not recommended in diabetic patients with severe renal dysfunction such as eGFR < 30 mL/min/1.73 m^2^. Therefore, the inclusion criteria of the present trial align with the official indications for ipragliflozin.

In summary, the PROCEED trial is the first to assess the effects of ipragliflozin on endothelial dysfunction in patients with T2D and established CKD. It is also intended to assess the potential effects of ipragliflozin on other cardiovascular and renal functional markers and the morphological changes in the pericardiac, hepatic, and perirenal fatty tissue and renal size measured by non-contrast abdominothoracic CT. It is believed that this trial will provide profound and novel insights into the putative underlying mechanisms of the renal benefits of SGLT2is in a patient population presenting with T2D and established CKD.

## Supplementary information

**Additional file 1.** Blood and urine examination.

**Additional file 2.** Trial organization.

## Data Availability

Not applicable.

## Abbreviations

AE: Adverse event
AI: Augmentation index
ANCOVA: Analysis of covariance
BP: Blood pressure
BW: Body weight
CI: Confidence interval
CKD: Chronic kidney disease
CRB: Certified review board
CT: Computed tomography
CVD: Cardiovascular disease
CVOT: Cardiovascular outcome trial
eGFR: Estimated glomerular filtration ratio
ESRD: End-stage renal disease
FAS: Full analysis set
HRV: Heart rate variability
LF/HF: Ratio of low-to high-frequency
LnRHI: Natural log-transformed RHI
LSM: Least square mean
MACE: Major adverse cardiovascular event
PR: Pulse rate
RAAS: Renin–angiotensin–aldosterone system
RHI: Reactive hyperemia index
RH-PAT: Reactive hyperemia- peripheral arterial tonometry
SDNN: Standard deviation of the normal to normal intervals
SGLT2i: Sodium glucose co-transporter 2 inhibitor
T2D: Type 2 diabetes
UACR: Urine albumin-to-creatinine ratio
